# *Prunus mira* Koehne in Sichuan, China: Recorded History as a Medicine and Food, Modern Applications, Distribution, and Ethnobotanical Investigations

**DOI:** 10.3389/fphar.2022.826712

**Published:** 2022-03-09

**Authors:** Jingwen Zhang, Wanyue Chen, Weijun Sun, You Zhou, Xiaoli Li, Jing Zhang, Gang Fan, Hongxiang Yin, Ju Qin, Yongcui Yuan, Wei Xu, Zhang Wang

**Affiliations:** ^1^ College of Pharmacy, Chengdu University of Traditional Chinese Medicine, Chengdu, China; ^2^ College of Ethnomedicine, Chengdu University of Traditional Chinese Medicine, Chengdu, China; ^3^ College of Medical Information Engineeringe, Chengdu University of Traditional Chinese Medicine, Chengdu, China

**Keywords:** *Prunus mira* Koehne, medicinal record history, botanical charateristics, modern research and application, ethnobotanical investigation, medicinal and edible, traditional Chinese medicine

## Abstract

*Prunus mira* Koehne, a *Prunus* plant in the Rosaceae family, is named ཁམབུ། in Tibetan and “Guang he tao” in Chinese. It is mainly distributed in Tibet Autonomous Region, Yunnan Province, and Sichuan Province in China. It is also a rare “living fossil group” of peach genetic resources in the world. It is used in traditional Chinese medicine for the treatment of dysmenorrhea, injury, intestinal dryness, constipation, and other diseases, and is used in Tibetan medicine for the treatment of hair, eyebrows, and beard shedding. In this article, the botanical characteristics, medicinal history, modern applied research, and ethnobotanical investigation of *P. mira* were recorded and evaluated. *P. mira* was first recorded in *Dumu Materia Medica*. *P. mira* in Sichuan Province is mainly distributed in Ganzi Tibetan Autonomous Prefecture, and has certain economic and medicinal value. *P. mira* has high nutritional composition. It is made into high-quality edible oil, cosmetic base oil, fruit juice, fruit wine, fruit vinegar, “Liang guo”, and other products. Oleic acid and linoleic acid are the main fat-soluble components of *P. mira*, which has an anti-inflammatory medicinal value and promotes hair growth. Its longevity and cold resistance can bring great genetic value and play an important role in maintaining peach genetic diversity. At present, there are few studies on the pharmacological effects of specific active components of *P. mira* and there are also few clinical studies. We can continue to study these aspects in the future. At the same time, products of *P. mira* have great market potential. All in all, *P. mira* is very worthy of further research and development.

## Introduction

*P. mira*, a *Prunus* plant in Rosaceae, is also known as *Amygdalus mira* (Koehne) Ricker. The Tibetan name is ཁམབུ། (transliteration for: Kangbu, Ximu, Kangkang, Shukan) (Gawu, 1995). The Chinese name is “Guang he tao”, “Maotao”, and “Tibetan peach”, because its core is smoother than *Prunus persica* (L.) Batsch, and so it is named *P. mira*. It is not only the original variety of wild peach in Tibetan areas of China, but also one of the most widely distributed varieties of wild fruit tree germplasm resources in Tibetan areas of China. *P. mira* has excellent characteristics such as strong cold resistance, drought resistance, barren resistance, disease resistance, and high concentration CO_2_ resistance (Ji et al., 2019), and its life span can reach thousands of years. It is the “living fossil group” of rare and precious peach germplasm resources in China and the world (Tan et al., 2012). *P. mira* is the most widely distributed peach in Tibet and has a high ornamental value. Because of the high elevation of *P. mira*, when the flowers of *P. mira* are in full bloom, the mountain opposite the forest is still covered with snow, and the scenery of the flowers of *P. mira* reflecting the snow-capped mountains opening is beautiful. Its fruit has a unique flavor and a high sugar content, which can be processed to make juice. The kernel of *P. mira* was included in *Sichuan traditional Chinese Medicine Standard* (1992 edition) (Health, 1991) and was used in traditional Chinese medicine to treat amenorrhea, dysmenorrhea, fall injury, intestinal dryness, and constipation. *Jingzhu Materia Medica* (1735) of Tibetan medicine (Tamar, 1986) and “Zang yao zhi” (1991) (Northwest Institute of Plateau Biology, 1991) recorded “seed extraction, oil and rub to treat yellow water disease, hair, eyebrow, beard and other prolapse”; now, it is also included in *the Standard of Tibetan Medicinal Materials of Sichuan Province* (2020 Edition). According to the “Quality arrangement and quality research of commonly used Chinese Medicinal Materials” (1997), Xu and Xu (1997) recorded that *P. mira* is distributed in the border areas of Sichuan Province, Yunnan Province, and Tibet Autonomous Region, mainly distributed in Batang County, Derong County, Xiangcheng County, Daocheng County, Yajiang County, Li County, Markang County, Jinchuan County, Muli County, and other counties in Sichuan Province; Zhongdian County and Ninglang County in Yunnan Province; and Bomi County, Chayu County, Mangkang County, Jiangda County, Basu County, and other counties in Tibet Autonomous Region. Because the book was published earlier, the distribution and reserves of *P. mira* recorded in the book may have changed greatly with the changes in society and the environment.

Ethnobotany is a comprehensive study of the relationship between botanical drugs in traditional ethnic and folk medicine and humankind in the region. It contains all plants that are culturally and economically important (Pei, 1988). The concept of ethnobotany was first put forward by Professor Hashburger, a botanist at the University of Pennsylvania in 1896 (Sun and Sheng, 2004). The purpose is to collect, screen, and seek the raw materials needed for industrialization through relevant knowledge of national plants (Pei, 2008). In the past 50 years, ethnobotany has paid more attention to the important knowledge in biodiversity conservation and sustainable utilization of native plants (Bhattarai et al., 2010), and the research field has also changed from resource development to traditional knowledge such as biodiversity conservation and sustainable utilization of plant resources (Pei, 2003). It is important to study traditional ethnic medicine at present. It has important theoretical value and practical significance to promote the inheritance of plant resources, diversity and characteristic culture in ethnic minority areas, and the sustainable development of regional economy and society (Pei, 2011). Kunming Institute of Botany, Chinese Academy of Sciences, the first institution specializing in ethnobotany in China, was established in 1987; the second international national biology conference was held in Kunming in 1990, which marked the preliminary launch of ethnobotany research in China (Li and Long, 2019). Tibetan areas of Sichuan are not only vast in territory, but also rich in plant resources. Its higher plants account for about one-third of China’s total, second only to Yunnan Province in China. The traditional Chinese medicine produced in the province accounts for 1/3 of the total output of traditional Chinese medicine in China, and it is the largest base of traditional Chinese medicine in China (Committee, 2018). Sichuan Province is located in southwest China; it borders Yunnan Province and Tibet Autonomous Region, and is the second largest Tibetan area in China, mainly including Ganzi Tibetan Autonomous Prefecture, Aba Tibetan and Qiang Autonomous Prefecture, and Muli Tibetan Autonomous County and Liangshan Yi Autonomous Prefecture, accounting for 51.49% of the total area of Sichuan Province (Census Office of the State Council, 2012). The ethnic medicinal plants in Sichuan Tibetan areas have a diverse variety, have wide applications and national characteristics, and have an important utilization and development value (Qiu, 2020). Among them, the Tibetan medicine *P. mira* is mainly produced in the Tibetan area of Sichuan, China. However, there are few ethnobotanical studies on Sichuan Tibetan areas, especially Tibetan medicine ethnobotany.

The data on the distribution of *P. mira* resources have been recorded for more than 30 years. During this period, due to pests and diseases (Xiang et al., 2019), social changes, climate and environmental changes, farmland reclamation, and other reasons, *P. mira* is under threat, and the natural population area of *P. mira* continues to decrease (Tang, 2012; Fachun et al., 2014; Tian et al., 2015). In a survey, it was found that about 90% of Tibetan residents knew *P. mira*, but more than half of Tibetan residents did not know it is a protected plant (Wang et al., 2021). At the same time, the main distribution area of *P. mira* has the tradition of raising yaks and Tibetan pigs, so the fruit is eaten in large quantities when it is ripe, and the wild *P. mira* tends to be aging seriously (Fang et al., 2008). Therefore, it is particularly important to conduct in-depth scientific and detailed protective research on *P. mira*.

With more in-depth medical research and the expansion of people’s cognition of ethnobotanical drugs, more and more scholars have carried out research on *P. mira*. Therefore, this paper expounds the medicinal record history, botanical characteristics, modern research and application, and ethnobotany research in Sichuan Province, to further develop the potential value of *P. mira* and provide reference for future research.

## Medicinal Record History of *P. mira*

The Tibetan medical works *Dumu Materia Medica* (750) (Xi, 2016) and *Gan lu ben cao ming jing* (1993) (Gama, 1993) provide the earliest records of *P. mira*. Their shape descriptions are that the leaves are lanceolate and the flowers are white, providing clues for the identification of *P. mira*. *Dumu Materia Medica* records that “Kangbu” was born in gullies, plains, and other places. Its trunk is tall and hard, with leaves like willow leaves. Its flowers are white and the fruit is red when ripe. According to *Gan lu ben cap ming jing*, “Kangbu” is a small perennial tree with purple stems, and hard and many branches; the new leaves are green and soft, the back of the leaf is light-colored, the leaves are alternate and lanceolate, the apex is long and pointed, the margin is serrated, and the petiole is long; the flower is pink with many petals. In spring, they grow leaves and then bloom. The summer fruits are green with yellow short hairs on the surface, and they become purple-red when they are mature. *Jingzhu Materia Medica (*1735) (Tamar, 1986) separates apricot and peach and describes them as two different plants; almond oil has the effect of curing hair loss, while semen persicae is used to ward off evil spirits, remove poison, and clear the throat, but has no effect on hair loss. In contrast, cores of “Zang tao” and “Kang mu tao” are smooth, which need to be further compared. *Jingzhu Materia Medica* (1735) reveals that apricot is divided into three kinds: “Shan xing” and “Chuan xing”; “Chuan xing” is further divided into “Han xing” and “Zang xing”. “Shan xing” tastes bitter, and “Chuan xing” tastes sweet. “Han xing” is large and sweet. “Zang xing” is inferior in flavor to “Han xing”, and its core is smooth. Almond oil promotes hair growth and darkens hair. There are three kinds of semen persicae: “Ru tao”, “Ci tao”, and “Kang mu tao”. There are four, five, and six joint seams on the surface of the core of the “Ru tao”. The core of “Ci tao” is like the *Phyllanthus emblica* L.; the surface has an abrupt grain. “Kang mu tao” is smooth and looks like the fruit of *Quercus robur* L. According to the *Zang yao zhi* (1991) (Northwest Institute of Plateau Biology, 1991), the Tibetan medical medicine “Kangbu” should belong to two plants of the Rosaceae family, and it involves two categories, namely, “Shan sheng” and “Chuan sheng”. “Shan sheng” includes *Prunus persica* (L.) Batsch and *P. mira*, which tastes bitter and is used in treating hair loss. “Chuan sheng” is divided into two kinds, which are produced in India and in Tibet, China, and has a sweet taste. In *Chinese Materia Medica* (1999) (Tibetan Medicine Volume) (Editorial Board of Chinese Materia Medica, 2002), it is believed that “Kangbu” has two kinds of Rosaceae plants: *P. mira* and *Prunus persica* (L.) Batsch. The kernel of *Prunus persica* (L.) Batsch is capable of treating hair loss, but the effect of the kernel of *P. mira* has not been recorded. According to Tibetan medicine, *P. mira* is generally called “Kangbu” and the *Prunus persica* (L.) Batsch is called “Kangburexia”. The fruit of *P. mira* is green with yellow fluff and turns reddish purple when ripe. The kernel of *P. mira* can promote hair growth and make the hair black, treat grasserie, hair, eyebrows, and other shedding disease.

Both *Chinese Materia Medica* (Tibetan Medicine Volume) and *Zang yao zhi* believe that *P. mira* belongs to “Kangbu”, and it may be that “*Chinese Materia Medica*” (Tibetan Medicine Volume) agrees with the view of *Zang yao zhi*. Through the textual research of these two herbs, it can be concluded that *P. mira* belongs to “Kangbu”, which has a therapeutic effect on alopecia, but it needs further identification. *Sichuan Standard of Chinese Medicinal Materials* (supplement) (Health, 1991) includes the dried and mature seeds of the *P. mira* of the Rosaceae family. After ripening, the seeds of *P. mira* were collected; the pulp and shell were removed and then dried. It can be used for amenorrhea, dysmenorrhea, lumps, bruises, intestinal dryness, and constipation. It is not recorded for hair loss. In *Newly revised Jingzhu Materia Medica* (Luo, 2004), it is believed that “Kang mu tao” is the original plant of today’s semen persicae, and the kernel oil of “Kang mu tao” has the effect of curing alopecia. According to the *Newly revised Jingzhu Materia Medica,* there are three kinds of peach: “Ru tao”, “Ci tao”, and “Kang mu tao”. The first two are not seen in today’s varieties, and the “Kang mu tao” is the original plant of today’s *Prunus persica* (L.) Batsch. There are three kinds of basic sources: *Prunus persica* (L.). Batsch., *P. mira*, and *Prunus davidiana* (Carrière) Franch, the kernel oil of all of which can promote hair growth and darken hair. According to the *Dictionary of Chinese ethnic Medicine*, *P. mira* is a plant of the Rosaceae family (Jia and Zhang, 2016). The oil extracted from seeds can cure baldness, and eyebrows and beard shedding, and the ash from pulp, core, and seed burning can cure various wounds, grasserie, and constipation. *The Standard of Tibetan Medicinal Materials of Sichuan Province* (2020 edition) includes *P. mira*, but uses its synonym (*Amygdalus mira* Koehne Yü et Lu). This standard recognizes the role of kernel of *P. mira* in the treatment of hair loss and establishes a relatively high-quality standard.

According to the above herbal authentication, it can be seen that different ancient Tibetan medical books have different understandings of the base source of “Kangbu”, which may be due to the complex geographical environment and the communication constraints in Tibetan areas. Both *Zang yao zhi* and *Chinese Materia medica* (Tibetan Medicine Volume) recognize *P. mira* as one of the sources of Tibetan medicine “Kangbu”. In addition, traditional Chinese medicine mainly uses the kernel of *P. mira* to treat gynecological and traumatic diseases, while Tibetan medicine mainly uses it to treat hair loss diseases, which expands the clinical application of the kernel of *P. mira*. However, the clinical experience and literature records have not been widely promoted.

## Botanical Characteristics of *P. mira*

### Distribution and Genetic Diversity of *P. mira*

*P. mira* is mainly born in coniferous and broadleaved mixed forest or hillside forest edge at 2,600–4,000 m altitude. It is highly adaptable, drought-tolerant, and light-loving and grows rapidly in favorable habitats. It is distributed in 20 counties between 31°10′-29°58′ north latitude and 91°50′-98°48′ east longitude, especially in the lower reaches of Yarlung Zangbo River and its tributaries Niyang River and Parlung Tsangpo River basin (Dong, 1991). It is produced in Sichuan Province (Batang County, Derong County, Xiangcheng County, Daocheng County, Yajiang County, Li County, Markang County, Jinchuan County, Muli County, and Yanyuan County), Yunnan Province (Shangri-La city, Deqin County, Ninglang County, etc.), Tibet (Bomi County, Mangkang County, Jiangda County, Badu County, and other counties) (Xu and Xu, 1997), and also in Nepal (Online, 2021). Zhou et al. (Zhou et al., 1994; Zhou et al., 1995) of the National Crop Germplasm Resources Investigation Team investigated the traces of *P. mira* in the study of rare core fruit trees in southwest Sichuan. They proposed that the kernel of *P. mira* could be divided into smooth type, shallow groove type, and deep groove type, and found that the three types of *P. mira* varied with the altitude. There are many types of variations of *P. mira* in Muli County, Yanyuan County and Mianning County of Sichuan Province. The annual average temperature for *P. mira* is 6–14°C. The average temperature of the coldest month (January) is −2.7°C, and the average temperature of the hottest month (July) is 18–19°C. It mainly grows in alpine shrubs and brown forest soil, alpine cultivated meadow soil, etc., and the soil quality is sandy loam, light sandy soil, or loam. The pH value of the land is between 6.4 and 7.5, and the habitat belongs to the sub-humid and semi-arid type (Zhong, 2008). Dong Guozheng made a preliminary investigation on *P. mira* resources in Tibet and estimated that there were about 3 × 10^5^ trees of *P. mira* in the whole region. The area was about 150–200,000 mu, and the annual yield of kernel of *P. mira* is about 5,000 kg (Dong, 1991). Only in the Nyingchi area of Tibet was the annual output about 5 × 10^6^ million kg (Cai et al., 1997a), and the total reserves were 53 × 10^5^−69 × 10^5^ kg (Zhong et al., 2010) according to the harvest year. However, due to farmland reclamation and overgrazing, the genetic diversity of *P. mira* was destroyed, and the area of *P. mira* in natural population was decreasing. Therefore, it is important to strengthen the resource conservation of *P. mira* in Tibet.

### Cultivation of *P. mira*

At present, the aging of *P. mira* is a serious problem in Nyingchi, Tibet. In field investigation, the regeneration of the seedlings in the native forests is rarely recorded (Fang et al., 2008). Scholars’ research on artificial cultivation of *P. mira* can provide a lot of seedling data for the cultivation of local *P. mira*. The seeds were treated with different concentrations of gibberellic acid (GA3), 6-benzyladenine (6-BA), and NaCl, and the effects on seed germination were observed. It was found that GA3, 6-BA, and NaCl could improve the germination rate, germination potential, and germination index of seeds of *P. mira* (Zhang et al., 2011). Geng et al. conducted a study on the seedling cultivation technology of *P. mira* (Geng et al., 2008). It was shown that the sowing and seedling of *P. mira* are relatively easy to operate, and the reproductive rate of *P. mira* can be effectively guaranteed. Seeds of *P. mira* had a higher germination rate after treatment, and asexual reproduction of *P. mira* can be carried out. The stem segments of the *P. mira* were cultured *in vitro* to study the effects of different sampling times, different sterilization methods, different media, different hormone types and concentrations on germination, axillary bud proliferation, and rooting. The results showed that the proliferation coefficient of the *P. mira* was higher under LP+6-BA 1.0 mg/L + IBA 0.2 mg/L. Rooting condition was 1/2 ms + IAA 1.0 mg/L + NAA 1.0 mg/L (Li et al., 2009). Li et al. studied the grafting technology of *P. mira* in Lhasa, Tibet, and discussed the best time for grafting of *P. mira* in a solar greenhouse. It was found that the best grafting period was from June 30 to July 10, and either too early or too late was not good for germination (Li et al., 2010). The effects of basic culture medium, sampling time, and disinfection method and plant growth regulator on the germination, proliferation, rooting, and transplanting of the bud stem segments of *P. mira* were studied. The results showed that the bud stem segments of *P. mira* were disinfected with 75% alcohol for 20 s and 0.1% mercury for 13 min on MS culture medium, and the bud stem segments of *P. mira* were disinfected with 75% alcohol for 20 s and 0.1% mercury for 13 min. Yellow loam:humus = 1:2 under transplanting matrix optimal conditions (Gao, 2019). In semi-arid areas such as Lhasa, Tibet, wood inlaid bud technology, peach t-shaped bud technology, and pipe protection technology have also been applied to achieve better adaptability of trees of *P. mira* (Mima et al., 2018).

### Morphological Characteristics of *P. mira*

**FIGURE 1:**
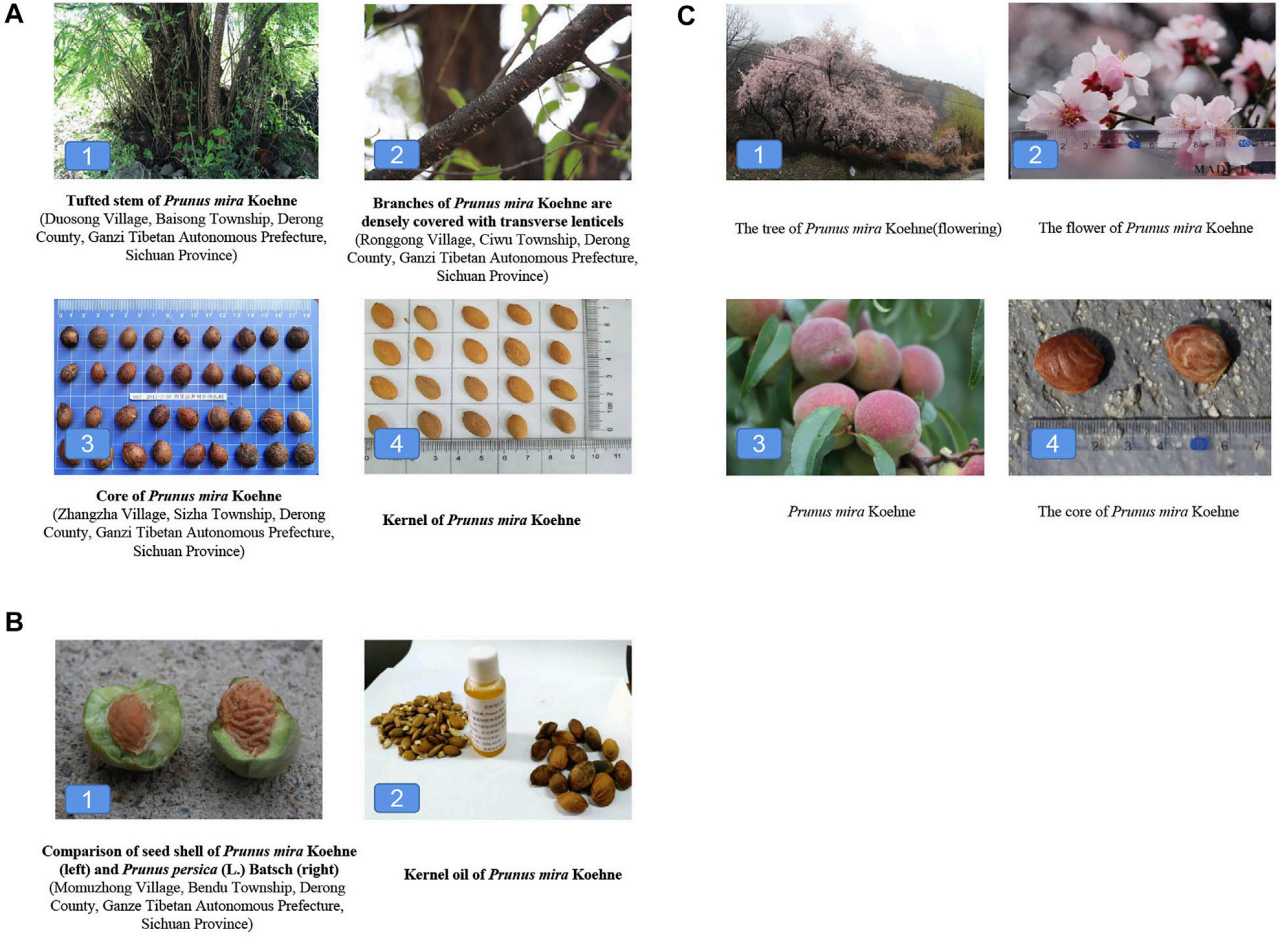
Morphological characteristics, identification characteristics, and ethnobotany of *P. mira* [**(A)**. Morphological characteristics of *P. mira*; **(B)**. Identification characteristics of *P. mira*; **(C)**. The tree, flower, fruit, and core of *P. mira*].

*P. mira* is a small deciduous tree; the tree height is 15–20 m, and the crown is 20–30 m. The root system is more developed (see Figure 1A for details). The diameter at the breast of the tree is 40 cm at 1 m above the ground; there are many transverse lenticels. Branchlets and buds have no villi or spines. Shoots are green and grayish brown when old with purplish-brown lenticels, but few lenticels are found on flowering branches (see Figure 1A for details). Leaf is blade lanceolate or ovate-lanceolate, 10–11.2 cm long, 2.5–3.6 cm wide, apex long acuminate, base obtuse or broadly cuneate, margin obtuse; leaf both surfaces glabrous or flanked tomentose on lower midvein surface; petiole is 1.2–1.3 cm long, apically with two to three glands, stipules are caducous. Rust-like insect pests are relatively less. The flowering period is from early March to mid-April, opening before the leaves; flowers are 3–3.5 cm in diameter, with short stems. Calyx is tube campanulate, glabrous, purplish red in color, 5 calyx lobes, ovoid, margin slightly pilose; 5 petals, white or pale pink, petal base pink, obovate, apex rounded obtuse, middle slightly concave. Some petals are not fully expanded and slightly overlapping. The stamens (about 41) are slightly longer than the style, not fully expanded, and the pistil is 1. The fruit ripening period is from mid-September to early October. It is suborbicular, densely fluffy, yellow green, and purple red on the sunny side, sour and juicy, with a long diameter of about 3.0–3.3 cm, a short diameter of about 2.9–3.2 cm, and a thickness of about 3.0–3.3 m; fruiting pedicel is about 4–5 mm long; the pulp and core are easy to separate. The core is oblate, hard, indehiscent, 1.9–2.1 cm long, 1.7–1.9 cm short, and 1.1–1.3 cm thick. The surface is yellowish brown, slightly compressed on both sides, asymmetrical, acute at the top, non-acute at the base, slightly oblique, with sharp ridges, smooth surface, only on the back and ventral surface with a few insignificant longitudinal shallow grooves, and no holes (see Figure 1A for details). The kernel is long oval or short oval, with a long diameter of about 1.3–1.5 cm, a short diameter of about 1.0–1.2 cm, and a thickness of about 0.4–0.5 cm. The surface is yellowish brown or brown, with fine granular protrusions. The tip is sharp, the base is blunt, slightly oblique, and the edge is thin. There is a linear hilum on one side of the tip, and most brown vascular bundle veins are scattered from the base chalazal, forming longitudinal concave lines covered with the seed coat. The seed coat is thin with 2 cotyledons, white in color, and oil-rich. The breath is slight and the taste is bitter and astringent, as detailed in Figure 1A.

The distinguishing feature of *P. mira* is that the kernel shell of this species is smooth (there are no holes on the surface of the kernel, with wide and shallow longitudinal grooves), which is easy to distinguish from other species (see Figure 1B for details). It is similar to *Prunus kansuensis* Rehder, but *Prunus kansuensis* Rehder has a cuneate leaf base, widest leaf above middle, and pilose outside calyx tube and sepal. There are also differences in distribution area.

### Other Biological Characteristics of *P. mira*

Under the special climatic conditions of long sunshine and strong radiation in Tibet, *P. mira* gradually evolved into having different photosynthetic characteristics from *Prunus persica* (L.) Batsch. In exploring whether high-intensity UV radiation can inhibit the photosynthesis and growth of *P. mira*, Hou et al. (2012) reached a preliminary conclusion after simulating the treatment of seedlings of *P. mira* with different intensities of UV-B radiation. In a short period of time, UV-B radiation can reduce the stomatal conductance and photosynthetic efficiency and then inhibit the growth of seedlings of *P. mira*. The annual average temperature in the distribution area of *P. mira* is 6–13°C, which is sensitive to high temperature. The growth and development of 1-year-old seedlings of *P. mira* were poor due to high temperature when they were transplanted in warmer areas of Beijing (the average temperature in summer is 25°C, with a high temperature of 37°C and an extremely high temperature of 42°C). The accumulation of ABA in leaves of seedlings of *P. mira* induced by HRT was increased, which may help to enhance the heat resistance of PSII (Hao et al., 2012). In recent years, most studies on the physiological characteristics of *P. mira* are focused on its photosynthesis, and the resistance of *P. mira* is also one of its important biological characteristics. Plant resistance refers to the evolution characteristics of plants in changing environments (climate, soil, water and nutrient supply, etc.). It is the result of adaptation to different environmental conditions. The research on plant resistance has an important guiding value for plant introduction and breeding. In recent years, the average annual temperature in Tibet has been gradually rising, the annual precipitation has been gradually declining, and the climate drought has become increasingly obvious (Smith, 2011). Then, drought has occurred frequently, which has brought great losses to the ecological environment and the economy in Tibet (Liu and Yuan, 2015). Drought stress is a common problem of plants. Some scholars have studied the effects of drought stress on seedlings of *P. mira* and analyzed the seedlings of *P. mira* in different soil moisture content of the protective enzyme activity and the change of photosynthetic characteristics, and it was found that under drought stress, to a certain extent, *P. mira* can increase water use efficiency; the soil moisture content is 10.1%–12.7%, and drought stress was the most significant (Guo et al., 2010). Under drought stress, both leaf (Cao, 2017) and root (Tian, 2016) had drought resistance and water retention ability, and all data were recovered following rehydration after severe drought, indicating that *P. mira* had strong drought resistance ability. By measuring the physiological indexes of *P. mira* under drought conditions, it was found that the drought resistance of *P. mira* was different with different provenances, among which shannan provenances of *P. mira* had a better drought resistance (Huang et al., 2018). However, studies have shown that the waterlogging resistance of *P. mira* is weak (Zhou et al., 2017; Zhang, Cuiling et al., 2019).

### Genetic Characteristics of *P. mira*

*P. mira* has excellent characteristics such as drought resistance, disease resistance, and longevity. It has high ecological, economic, and ornamental value. It has made a lot of contributions to promoting the evolution of peach resources and cultivating new varieties with high resistance. The product value, intellectual property, and patent value of *P. mira* to be developed will be considerable. Under the influence of many factors, the existing resource development and research are not enough, and the research on the protection and sustainable utilization of its genetic resources still needs more effort (Zhang et al., 2013). Most studies have focused on the ecology and genetic diversity of *P. mira* (Bortiri et al., 2001; Fang et al., 2008). *P. mira* not only is considered as an important gene bank for improving germplasm resources of cultivated peach, but also can be used to control soil erosion and restore vegetation because of its high tolerance to harsh environments (drought, cold, and barren soil) (Hao et al., 2009). Because of its high tolerance to harsh environments and high yield, it has great potential in peach breeding (Li et al., 2014; Tian et al., 2015).

*P. mira* has a long life, is disease resistant, and has barren characteristics. Through research, it is found that the life span of *P. mira* is 5–20 times longer than that of ordinary peach species, and it has the potential to become one of the longest peach species. It can not only contribute to extending the life of other peach species, but also increase the output value of *P. mira* (Bao et al., 2018a). Because of its high disease resistance, *P. mira* can survive and produce better results under environmental stress, which can significantly reduce the capital investment in labor costs and pesticide costs. At present, the improper use of pesticides leads to the continuous decline of land quality, which has attracted great attention from all over the country. The improper use of chemical fertilizers (such as the use of excessive amount of nitrogen and phosphorus) also occurs in the peach-growing areas in China (Wang et al., 2009). Improper fertilization also restricts the development of the peach industry. At present, less than 1% of pesticides can be used when fruit trees are planted and sprayed, and most pesticides are wasted or remained on fruit, which is harmful to human health (Xue, 2019). *P. mira* grown on the plateau are rarely contaminated by pesticides. The resistance of different species and types of peach to root-knot nematodes was determined through years of natural disease nursery sowing and pot experiments, and it was found that some *P. mira* have the potential to become resistant (Zuo et al., 1988).

*P. mira* has important genetic resource value. Resources of *P. mira* have the value of primitive species. Some scholars have determined that *P. mira* is the original species of peach resources through studies on its distribution and morphological changes of fruit cores. It is produced in Tibet, northwestern Yunnan Province, western Sichuan Province, and other places in China, and the above areas are the origin centers of all peach species (Wang and Zhou, 1990). At present, the genetic evaluation of peach germplasm resources was carried out from the aspects of morphology, SSR marker, palynology, isoenzyme, RAPD marker, etc., and some conclusions about the occurrence and phylogenetic evolution of peach population were obtained. Through electron microscope scanning of pollen grains, Guo et al. found that the pollen grains of *P. mira* were elliptical in shape, and the outer wall feature had simple parallel straight stripes without perforation, and it was the most original wild species of peach subgenus (Guo et al., 2006). The pollen morphology of five Tibet wild *P. mira* was observed using an scanning electron microscope, the pollen morphology diversity of four petal variant materials was significant, and the pollen patterns of the five materials were all primitive (Zeng, 2016). Zhou analyzed the anther water-soluble proteins of *P. mira*, *Prunus kansuensis* Rehder, Prunus davidiana (Carrière) Franch, and *Prunus persica* (L.) Batsch by isoelectric aggregation (IEF). It was found that *P. mira* had the simplest protein band and showed primitiveness (Zhou et al., 1998). Zongxue et al. analyzed the pollen protein by sodium dodecyl sulfate (SDS) electrophoresis. It is also believed that the primordiality of *P. mira* is the strongest (Zong et al., 1995). In order to provide basic genetic resources for comparative population genomics studies, the complete chloroplast genome sequence of *P. mira* was studied (Amar et al., 2018). The complete chloroplast (Cp) genome is assembled from scratch using low-coverage whole genome sequencing data, and phylogenetic analysis showed that *P. mira* was the most primitive and basic lineage in the subgenus *Pmygdalus* (subfamily *Prunoideae*), consistent with traditional classification (Bao et al., 2019). Genetic diversity is the basis for long-term survival and evolution of species (Nei, 1978). Wild *P. mira* has rich genetic diversity and great potential for exploitation and utilization. At the second International Biodiversity Ecology and Environment Conference in 2013, some scholars aimed to use simple sequence repeat (SSR) technology to study the diversity and genetic relationship of peach species such as *P. mira*, and provided experience for improving breeding programs. Thus, the economic value and ornamental value of *P. mira* can be improved (Xing et al., 2013). The genetic diversity level of *P. mira* should be consistent with the characteristics of wide distribution, strong adaptability, and high genetic diversity level of outcrossing species believed by most scholars (Liu et al., 2012). Two molecular marker methods (SSR and ISSR) are used to study 72 materials. The results show that there is a certain correlation between geographical location and genetic distance. Wild *P. mira* and “Henan tao” are closely related, so *P. mira* could be considered as a resource for the development of peach germplasm, which provides a theoretical basis for the development and protection of peach germplasm resources to a certain extent (Xing, 2014). Amplification fragment length polymorphism (AFLP) was used to analyze the genetic relationship of 83 germplasm resources of *P. mira* from five populations, and it was found that the genetic diversity of germplasm resources of *P. mira* was high, which provided a new gene source for peach rootstock variety breeding (Li et al., 2014). Bao et al. used SSR markers to deeply study the genetic diversity and genetic structure of *P. mira* in Tibet. It is found that the genetic diversity of *P. mira* was high, with genetic variation of geographical isolation and elevation gradient among different populations, and the level of genetic differentiation was relatively good (Bao et al., 2018b). Tan et al. used SRAP molecular marker technology to analyze the genetic diversity of natural populations of *P. mira* in Tibet, and clarified its genetic diversity level and genetic structure at the molecular level. It was found that *P. mira* had a higher level of genetic diversity, less genetic differentiation among populations, and a higher degree of gene communication among populations (Tan et al., 2012). According to domestic and foreign research materials, *Prunus amygdalus* Batsch can be crossed with *Prunus persica* (L.) Batsch, *P. mira*, *Prunus davidiana* (Carrière) Franch, and other resources; many hybrid offspring have characteristics such as stress resistance, and *P. mira* has provided its own genetic characteristics (Ma et al., 2002).

## Modern Research and Application of *P. mira*

### Chemical Composition

There are many studies on the chemical constituents of the kernel of *Prunus persica* (L.) Batsch and the kernel of *Prunus davidiana* (Carrière) Franch in the *Pharmacopoeia of the People’s Republic of China*, but there are few studies on the chemical constituents of the kernel of *P. mira*. According to the comparison of HPLC fingerprints of the kernel of *P. mira* and the kernel of *Prunus persica* (L.) Batsch, it was found that the chemical components of the kernel of *P. mira* and the kernel of *Prunus persica* (L.) Batsch were similar (Du et al., 2019). After the analysis of seed oils of six plants in Tibet, it was found that the main fatty oils of *P. mira* were myromytic acid (0.1%), palmitic acid (7.4%), palmitoleic acid (0.3%), stearic acid (2.8%), oleic acid (60.7%) and linoleic acid (28.7%), and the main fatty acid component of the seed oil of *P. mira* was oleic acid (Zhang et al., 1979). After optimizing the extraction process of the kernel oil of *P. mira*, it was found that it had excellent physical and chemical properties, and 12 kinds of fatty acid components were detected; the main fatty acids were cis-oleic acid (57.32%), linoleic acid (31.65%), palmitic acid (6.49%), and stearic acid (2.29%), and unsaturated fatty acids (89.43%) were the main fatty acids. Among them, polyunsaturated fatty acids (31.76%) and monounsaturated fatty acids (57.67%) were the main types (Kan et al., 2020). Some studies had shown that oleic acid and linoleic acid are the main fatty acids in *Prunus* plant in the Rosaceae family (Kodad and Socias I Company, 2008; Sathe et al., 2008). Fatty acid composition of the kernel of *P. mira* from different provenations in Tibet was analyzed, and it was found that unsaturated fatty acid content of the oil of *P. mira* from different regions in Tibet was more than 90% (Wang and Wei, 2016), which was mainly oleic acid and linoleic acid, similar to the results of previous studies (Wei et al., w). Wei et al. conducted qualitative and quantitative analysis on the fatty acids of the oil of *P. mira* in Nyingchi, Tibet. The oil yield of the kernel of *P. mira* was as high as 51.4%, and the content of unsaturated fatty acids was 91.98%. Its main components were oleic acid and linoleic acid, while the main components of saturated fatty acids were palmitic acid and stearic acid. The fat-soluble components of different varieties of semen persicae were identified by the GC-MS method. It was found that there were significant differences in fat-soluble components of different varieties and origins of semen persicae, among which there were 16 chemical components (benzaldehyde, trans-2,4-decadienal, tedocane, cyclododecene, 8-heptatene, Ethyl palmitate, methyl oleate, oleic acid, 9,17-octadecadienal, linoleic acid, methyl linoleate, squalene, β-tocopherol, Vitamin E, β-sitosterol, and 1-eicosene) of fat-soluble components in the kernel of *P. mira* (Liang et al., 2012). Sun et al. (2018) analyzed the fat-soluble components of the kernel oil of *P. mira* by GC-MS, and the 35 compounds of the kernel oil of *P. mira* were mainly oleic acid, β-sitosterol, trans-squalene, γ-tocopherol, and vitamin E. Then, the contents of vitamin E, squalene, β-sitosterol, and α-tocopherol were determined by HPLC (Liang, 2011). A total of 41 compounds were identified by GC-MS, mainly oleic acid, β-sitosterol, trans squalene, γ-tocopherol, and vitamin E, and an HPLC method was established for the determination of α-tocopherol, vitamin E, and β-sitosterol of the kernel oil of *P. mira* (Sun, 2018). In addition, HPLC method was used to establish a method for the determination of oleic acid, linoleic acid, and amygdalin of *P. mira* (Zhou, 2020). Compared with commercial peach, *P. mira* of Tibet is rich in polyphenols, flavonoids, polysaccharides, and other functional substances, and the polyphenol content (2,716.09 ± 14.10 mg GAE/100 g d.b.) was 3–5 times higher than that of commercial peach; monophenols mainly included catechin, chlorogenic acid, and neochlorogenic acid. The contents of flavonoids and polysaccharides were 2,179.21 ± 11.20 mg RE/100 g d.b. and 9,393.81 ± 284.97 mgGE/100 g d.b., respectively, which were about three times and two times that of commercial peaches (Zuo, 2019). *P. mira* is also rich in a variety of trace elements, and the contents of Cu, Fe, Mn, Zn, Ca, and Mg in the kernel of *P. mira* from 10 provenance areas in Tibet were determined by atomic spectrophotometry. The results showed that the mean value of trace element content among provenances was Mg > Ca > Zn > Fe > Mn > Cu, and the variation coefficient of trace element content among different provenances was Ca > Mn > Cu > Fe > Zn > Mg (Wei et al., 2017). The relevant information is shown in Table 1.

**TABLE 1:** Chemical composition of *P. mira*.

Plant part	Extraction method	Detect method	Main chemical compositions	References
The kernel of *P. mira*	Ethanol extraction	HPLC	Oleic acid, linoleic acid, D-Amygdalin hydrate	Du et al. (2019)
The kernel of *P. mira*	Petroleum ether extraction	Solvent reflux method	Myromytic acid, palmitic acid, palmitoleic acid, stearic acid, oleic acid, linoleic acid	Zhang et al. (1979)
The kernel of *P. mira*	Aqueous extraction	GC	Cis-oleic acid, linoleic acid, palmitic acid, stearic acid	Kan et al. (2020)
The kernel of *P. mira*	Petroleum ether extraction	Solvent reflux method, gas chromatography	Oleic acid, linoleic acid	Wang and Wei (2016)
The kernel of *P. mira*	Petroleum ether extraction	GC	Oleic acid, linoleic acid, palmitic acid, stearic acid	Wei et al. (2013)
The kernel of *P. mira*	Petroleum ether extraction	GC-MS	Benzaldehyde, trans-2, 4-decadienal, tedocane, cyclododecene, 8-heptatene, Ethyl palmitate, methyl oleate, oleic acid, 9,17-octadecadienal, linoleic acid, methyl linoleate, squalene, β-tocopherol, vitamin E, β-sitosterol, 1-eicosene	Laing et al. (2012)
The kernel of *P. mira*	Petroleum ether extraction	GC-MS	Oleic acid, β-sitosterol, trans-squalene, γ-tocopherol, vitamin E	Sun et al. (2018)
The kernel of *P. mira*	Petroleum ether extraction	HPLC	Vitamin E, squalene, β-sitosterol, α-tocopherol	Liang (2011)
The kernel of *P. mira*	Petroleum ether extraction	GC-MS	Oleic acid, β-sitosterol, trans squalene, γ-tocopherol, vitamin E	Sun (2018)
The kernel of *P. mira*	Potassium hydroxide ethanol extraction, methanol extraction	HPLC	Oleic acid, linoleic acid, amygdalin	Zhou (2020)
The fruit of *P. mira*	Methanol extraction, ethanol extraction	Vacuum freeze drying combined with ultrafine grinding technology	Polyphenols, flavonoids, polysaccharides, catechin, chlorogenic acid, neochlorogenic acid	Zuo (2019)
The kernel of *P. mira*	—	Atomic absorption spectrophotometer	Cu, Fe, Mn, Zn, Ca, Mg	Wei et al. (2017)

### Pharmacological Effects

The fat-soluble components of the kernel of *P. mira* have obvious anti-inflammatory and vasodilating effects (Xu and Xu, 1997). The kernel of *P. mira* (Liu et al., 1989) could significantly increase the flow of isolated rabbit ear vasoperfusion fluid, eliminate the vasoconstriction effect of norepinephrine, and also show significant anti-inflammatory effects on rat paw swelling caused by egg white. The LD50 of the kernel of *P. mira* decoction on mice was 42.81 ± 0.02 g/kg, which was 238 times the commonly used dose (0.18 g/kg) in clinical patients. The long-term toxicity test of rats showed no significant effect on hematology, blood biochemistry, and histopathology. In the acute toxicity test of *P. mira*, the maximum dose of the kernel oil of *P. mira* orally in rats and mice was 144.612 and 289.224 g crude drug/(kgd), respectively, and the maximum dose of the kernel oil of *P. mira* orally in rabbits was 482.28 mg crude drug/(cm^2^ d). It had good security (Sun et al., 2017). Two kinds of hair removal methods (sodium sulfide hair removal cream and hair removal instrument) were used to study the effects of different doses of the kernel oil of *P. mira* on hair growth in KM mice, and it was found that the kernel oil of *P. mira* could promote the transformation of hair follicles into growth stage and promote hair growth in mice (Zhou et al., 2020). The fat-soluble components of the kernel of *P. mira* can promote hair growth in the range of 15.06–60.26 mg/cm^2^/d (Sun, 2018), but the material basis of its efficacy is still unclear. The results showed that vitamin E (3.125 mg/cm^2^/d), β-glutenosteroid (0.061 mg/cm^2^/d), and linoleic acid (0.156 mg/cm^2^/d) could promote the hair growth of mice. Its mechanism may be related to the Wnt/β-catenin pathway (Zhou, 2020). Amygdalin from the kernel of *P. mira* was extracted by ultrasonic oscillation method, and different concentrations of amygdalin from the kernel of *P. mira* extract had different killing effects on different insects (slugs, cabbage worms, and aphids) (Yang et al., 2020). The relevant information is shown in Table 2.

**TABLE 2:** Pharmacological effects of *P. mira*.

Plant part	Extractive	Animal	Administration method	Animal model	Dose	Activity	References
The kernel of *P. mira*	Decoction	Wistar rat	Gastric irrigation	Foot swelling model	5.084 g crude drug/kg	Anti inflammation and vasodilation	Liu et al. (1989)
The kernel of *P. mira*	Oil	SD rat; KM mice; Rabbit	Gastric irrigation; Skin administration	Depilation model	144.612 g crude drug/(kg·d); 289.224 g crude drug/(kg·d); 482.28 mg crude drug/(cm^2^·d)	Good safety	Sun et al. (2017)
The kernel of *P. mira*	Oil	KM mice; C57BL/6 mice	Skin administration	Depilation model	Vitamin E 3.125 mg/cm^2^/d, β-sitosterol 0.061 mg/cm^2^/d, linoleic acid 0.156 mg/cm^2^/d	Promote hair growth	Zhou et al. (2020)
The kernel of *P. mira*	Amygdalin	Slugs, cabbage worms, aphids	—	—	10 g crude drug/L	Insecticidal	Yang et al. (2020)

### Product Development

*P. mira* resources are rich in Tibet, but due to its self-generation and self-extinction, the resources have not been fully utilized. Biological test method was used to analyze the contents of soluble sugars, organic acids, and mineral elements in batches of *P. mira* from different producing areas. Due to the differences in basic quality characteristics and physiological and biochemical characteristics of fruits of *P. mira* from different producing areas, most of them lean towards the general level (Liu and Meng, 2013). Many scholars take into account the taste characteristics of *P. mira*, so they explore the processing technology to improve the taste of their products. EDTA, citric acid, VB-Na, phytic acid, and NaCl were selected as composite preservatives for storage of pulp of *P. mira*. U12 (64 × 2) uniform design was adopted. The optimal comprehensive formula was obtained as follows: EDTA dosage was 0.002%, citric acid was 0.01909%, VB-Na was 0.04%, phytic acid was 0.019%, and NaCl was 0.159% (Zhong et al., 2014). Compared with ordinary *Juglans regia* L., the bitter taste of *P. mira* was obvious. The pulsed vacuum debitter process was used to remove 95.77 ± 0.116% amygdalin in wild kernel of *P. mira*, and can significantly shorten the debitter time and improve the edible ability of kernel of *P. mira* (Zhang, Chaoqi et al., 2019). The mineral and vitamin C content of *P. mira* is higher than that of ordinary peach, and its pulp is rich in pectin, cellulose, and dietary fiber. The potential of developing functional products of dietary fiber cannot be underestimated (Zeng et al., 2009). The commercialization of the fruit *P. mira* and its processed juice has produced considerable economic benefits. The traditional processing method is to make dried peach. At present, after *P. mira* was debittered and most of the crude cellulose was degraded, highly nutritious mixed juice and preserves with a suitable taste could be made, providing new ideas for the product development of *P. mira* in Tibet (Cai et al., 1997a). Three products such as “juice of *P. mira*”, “preserved fruit of *P. mira*” and “Liuhe” fruit tea of *P. mira* had also been successfully developed (Cai et al., 2002). In the trial production of mixed juice of *P. mira* in Tibet, it was found that 15% juice of *P. mira* and 5% apple juice could be mixed to obtain a mixed juice with a rich flavor (Cai et al., 1997b). Response surface methodology (RSM) was used to optimize the fermentation process of wine of *P. mira*. It was found that the optimal fermentation conditions of wine of *P. mira* were as follows: after juicing the fresh fruit of *P. mira*, the initial sugar content was adjusted to 18.84%, pH 3.96, the yeast inoculation amount was 0.76‰, and the volume fraction of alcohol was 11.33%. After fermentation and aging, the wine has a golden color and a mellow and sweet taste (Zhong et al., 2012). Some scholars took pectinase as a clarifier to determine the optimal clarification process parameters and obtain the clarified juice of *P. mira*, which laid a foundation for the development of the juice, wine, and fruit vinegar of *P. mira* (Kan et al., 2018). The liquid–solid string leaching fermentation process was used to obtain *P. mira* fruit vinegar with 86.4% alcohol fermentation rate, 75.3% acetic acid conversion rate, and a fermentation period of 15–16 days (Zhong and Fang, 2011). Many scholars have also explored the processing technology to ensure the antioxidant capacity of *P. mira*. Steam hot treatment could significantly improve the content of nutrients and antioxidant capacity of *P. mira*, which could be used as an effective pretreatment method for deep processing of *P. mira* (Zuo et al., 2018). The ultra-fine powder of *P. mira* with excellent quality could be prepared by vacuum freeze drying technology and ultra-fine grinding technology on the premise of retaining the main active components and antioxidant capacity of *P. mira* to a large extent (Zuo et al., 2019). “Liang guo” of *P. mira* (Zhong and Pu, 2001) was also a good product of *P. mira*. *P. mira* has high oil content and is mainly composed of oleic acid and linoleic acid. At the same time, it is rich in Ve and other active substances (Wang and Wei, 2016). It could be used as edible oil (Luo and Zheng, 1998), as well as cosmetics base oil and advanced lubricating oil. Flesh color is one of the important traits affecting the value of peach fruit products. The study on the main metabolites and transcripts related to peach fruit coloring provides useful information for the improvement of peach fruit quality and provides theoretical support for improving the attractiveness and quality of peach varieties.

## Investigation on Ethnobotany of *P. mira* in Sichuan Province

### Survey Methods

**FIGURE 2:**
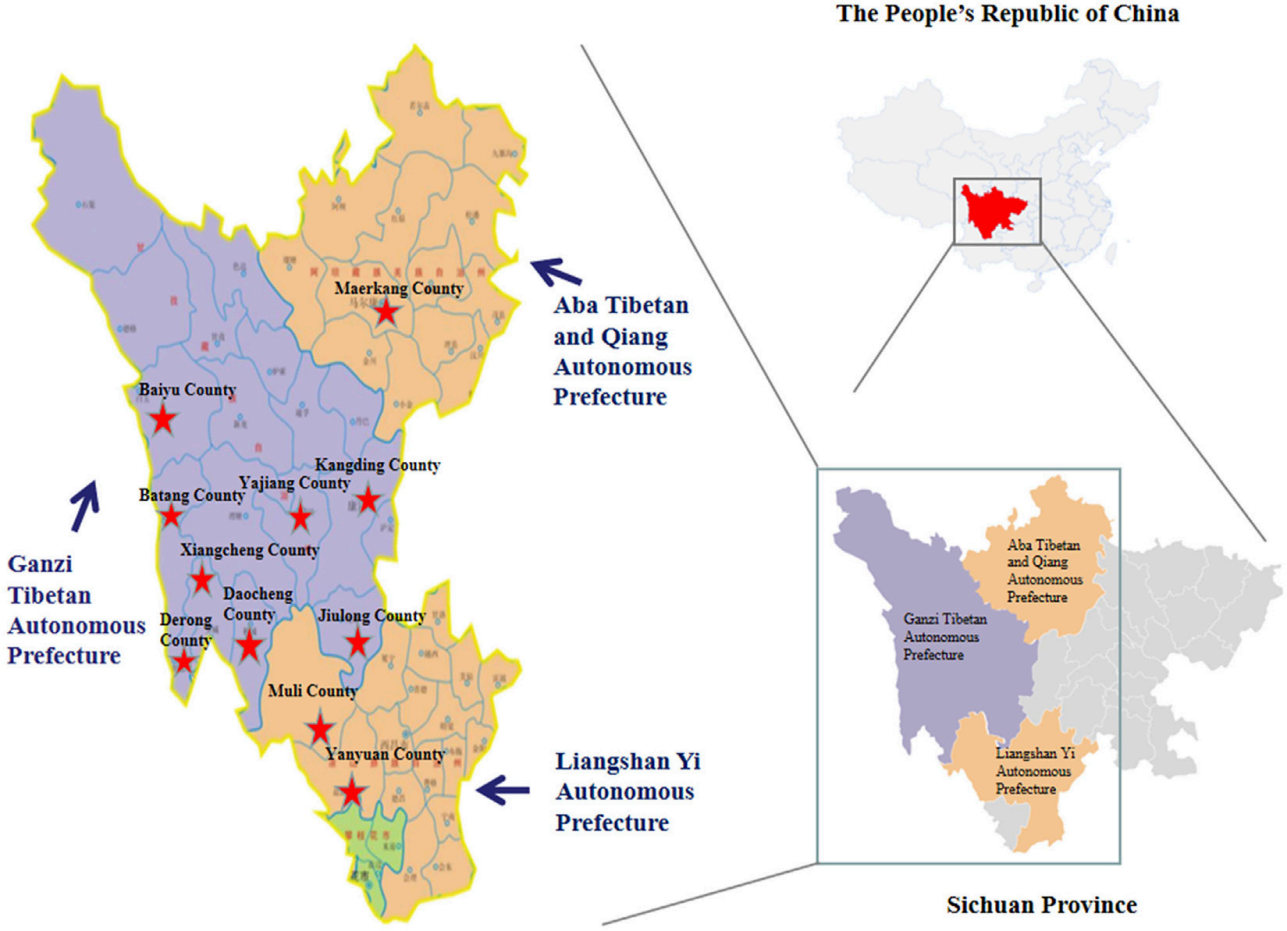
Distribution of survey sites. (★ The survey site is marked with red pentagonal stars in the figure)

China is in the east of Asia. Sichuan Province is located in southwest China and is the second largest Tibetan area in China. Sichuan Ganzi Tibetan Autonomous Prefecture, Aba Tibetan and Qiang Autonomous Prefecture, and Liangshan Yi Autonomous Prefecture covered most of the production areas of *P. mira*, so the counties and cities of these three prefectural regions were mainly selected for research (see Figure 2 for details). Books related to the history, identification, medicinal value, resource distribution, and usage of *P. mira* had been widely collected, including modern herbal works, medicinal materials standards, and flora and literature data. Meanwhile, researchers examined specimens at the Chengdu Institute of Biology, Chinese Academy of Sciences to determine the scientific name, origin, distribution, botanical characteristics, distribution area, and ecological environment of the *P. mira*. The geographical topography, climate environment, species distribution, and other data of the three prefectural counties along 318 National Highway, including Derong County, Xiangcheng County, Daocheng County, Batang County, Yajiang County, Markang County, Muli County, and Yanyuan County, were consulted to clarify the basic situation of the survey area and the possible growth area of *P. mira*. The second step was to interview key people. The researchers learned about the quantity, distribution, cultivation area, medicinal material yield, market price, and other information from local forestry bureau, agricultural bureau and other units, planting bases, farmers, and herdsmen, and further determined the investigation sites at township and village levels. The third step was field investigation and specimen collection. Through field investigation, the quantity, distribution, morphology, habitat, wild resources, planting situation, use situation, and market situation of *P. mira* in Sichuan Province were understood and recorded. At the same time, the plants were photographed and proof specimens were made for identification purposes.

### Basic Information

The relevant information of the surveyed areas is shown in Table 3. Specimens collected from various places were identified as *P. mira* by Professor Jia Minru of Chengdu University of Traditional Chinese Medicine (see Figure 1C for details). The condition of the tree of wild *P. mira* was recorded in detail, which included tree age, tree height, crown width, trunk diameter, leaf length and width, fruit, core and kernel length, width, and thickness, all quantifiable indicators. The results showed that *P. mira* mainly grew in 2,500- to 3100-m mountain valleys, mountain slopes, vegetable fields on the edge of fields, and on the front yard of houses. Because the national road and provincial road land utilization rate is higher, more trees of *P. mira* were cut down and the semi-high mountains and less inhabited forest more, meadow and pine forest is rarely seen *P. mira* distribution. The age of the tree of wild *P. mira* ranges from 15 to 100 years; the tree height is between 3 and 15 m, the crown diameter is between 5 and 15 m, and the circumference of the trunk 1 m above the ground is 50–500 cm. The flowering period is from March to April, and the fruiting period is from September to October. The leaf length is 5–15 cm, the width is 1.0–4.0 cm, the number of glands is 2–7, the fruit weight is 27.70 ± 13.29 g, the long diameter is 2.2–5.0 cm, the short diameter is 1.7–4.0 cm, and the thickness is 1.0–4.0 cm. The weight of the kernel is 1.32 ± 0.69 g, the average nucleation rate is about 10%, the long diameter of nucleus is 1.2–2.6 cm, the short diameter of nucleus is 1.0–2.0 cm, the thickness of the kernel is 0.8–1.7 cm, the weight of the kernel is 0.24 ± 0.13 g, the diameter of the kernel is 1.1–2.1 cm, the diameter of kernel is 0.7–1.8 cm, the thickness of kernel is 0.4–0.8 cm, and the average rate of kernel emergence is about 17%. Associated plants are *Juglans regia* L., *Fagopyrum esculentum* Moench, and so on. In the course of investigation, it was also found that the main mixed varieties of *P. mira* in the Tibetan areas of Sichuan were *Prunus kansuensis* Rehder and *Prunus tangutica* (Batalin) Koehne. A large number of *Prunus kansuensis* Rehder were found in Heishui County, Aba Tibetan and Qiang Autonomous Prefecture, and local farmers also called it “Mao tao”.

**TABLE 3:** Information and number of survey sites in Sichuan Province.

Number	Serial number	Autonomous prefecture	County	Township/Town	Village	Altitude (m)	Longitude	Latitude	Survey date
1	160518DRXY	Ganzi Tibetan autonomous prefecture	Derong county	Guxue township	Xiayong village	2,589	99°18′5706″	28°24′4128″	2016.5.18
2	160723DRMU	Ganzi Tibetan autonomous prefecture	Derong county	Bendu township	Momushang village	2,600	99°21′1667″	28°36′8444″	2016.7.23
3	160724DRMZ	Ganzi Tibetan autonomous prefecture	Derong county	Baisong township	Menza village	2,568	99°22′5640″	28°54′4644″	2016.7.24
4	160917DRDS	Ganzi Tibetan autonomous prefecture	Derong county	Baisong township	Duosong village	2,889	99°42′5068″	28°93′3574″	2016.9.17
5	160724DRLD	Ganzi Tibetan autonomous prefecture	Derong county	Ciwu township	Langda village	3,095	99°16′5692″	28°57′5104″	2016.7.24
6	160917DRRG	Ganzi Tibetan autonomous prefecture	Derong county	Ciwu township	Ronggong village	3,275	99°29′8785″	28°00′0438″	2016.9.17
7	170917DRKS	Ganzi Tibetan autonomous Prefecture	Derong county	Ciwu township	Kase village	2,783	99°20′1049″	28°55′1383″	2017.9.17
8	160724DRQY	Ganzi Tibetan autonomous prefecture	Derong county	Songmai town	Quya village	3,300	99°19′4115″	28°46′5876″	2016.7.24
9	160724DRZZ	Ganzi Tibetan Autonomous Prefecture	Derong county	Sizha township	Zhangzha village	3,000	99°15′3838″	28°48′5699″	2016.7.24
10	160724DRKG	Ganzi Tibetan autonomous prefecture	Derong county	Sizha township	Kalong village	2,500	99°17′13.38″	28°48′3599″	2016.7.24
11	160725DRRD	Ganzi Tibetan autonomous prefecture	Derong county	Rilong township	Ridui village	3,028	99°11′1158″	28°41′2357″	2016.7.25
12	160916DRRD	Ganzi Tibetan autonomous prefecture	Derong county	Rilong township	Ridui village	2,934	99°18′8960″	28°68′9485″	2016.9.16
13	170720DRLR	Ganzi Tibetan autonomous prefecture	Derong county	Rilong township	Longrong village	3,196	99°12′3309″	28°41′3902″	2017.7.20
14	170918DRLR	Ganzi Tibetan autonomous prefecture	Derong county	Rilong township	Longrong village	3,196	99°12′3309″	28°41′3902″	2017.9.18
15	160925DRZR	Ganzi Tibetan autonomous prefecture	Derong county	Xulong township	Zhangren village	2,935	99°15′7005″	28°74′1808″	2016.9.15
16	170401DRJL	Ganzi Tibetan autonomous prefecture	Daocheng county	Chitu township	Jiala village	3,164	100°16′1899″	27°37′3276″	2017.4.1
17	170916XCZD	Ganzi Tibetan autonomous prefecture	Xiangcheng county	Zhengdou township	Yongde village	2,750	99°31′2148″	29°05′4042″	2017.9.16
18	170916XCML	Ganzi Tibetan autonomous prefecture	Xiangcheng County	Dingbo township	Mala village	2,841	99°31′4347″	29°13′2911″	2017.9.16
19	170917BTDQ	Ganzi Tibetan autonomous prefecture	Batang county	Zhongzan town	Duoqiang village	2,929	99°19′1089″	29°21′2186″	2017.9.17
20	170917BTXB	Ganzi Tibetan autonomous prefecture	Batang county	Zhongzan town	Xuebo village	2,867	99°14′1627″	29°19′1000″	2017.9.17
21	170922KDCC	Ganzi Tibetan autonomous prefecture	Kangding county	Pusharong township	Changcaoping village	2,922	101°19′1446″	29°32′1447″	2017.9.22
22	170923YJWX	Ganzi Tibetan autonomous prefecture	Yajiang county	Bajiaolou Township	Wangjiayi village	2,719	101°06′1872″	30°06′0709″	2017.9.23
23	170924JLCE	Ganzi Tibetan autonomous prefecture	Jiulong county	Xiaer town	Chaer village	2,823	101°30′3892″	28°59′1512″	2017.9.24
24	180821BYJS	Ganzi Tibetan autonomous prefecture	Baiyu county	Jinsha township	Jisonggang village	2,924	100°48′1240˝	31°16′4831″	2018.8.21
25	170926MLTB	Liangshan Yi autonomous prefecture	Muli county	Wachang town	Taoba village	2,577	100°50′2772″	28°09′4138″	2017.9.26
26	170927MLWJ	Liangshan Yi autonomous prefecture	Muli county	Wujiao township	Wujiao village	2,754	100°44′4022″	27°57′5178″	2017.9.27
27	170927YYDL	Liangshan Yi autonomous prefecture	Yanyuan county	Qiansuo township	Doule River	2,559	100°47′2188″	27°52′1083″	2017.9.27
28	170927YYDZ	Liangshan Yi autonomous prefecture	Yanyuan county	Luguhu town	Dazu village	2,636	100°47′0130″	27°45′1395″	2017.9.27
29	180611MEKS	Aba Tibetan and Qiang autonomous prefecture	Markang county	Songgang town	—	2,531	102°06′0027″	31°55′1044″	2018.6.11
30	180614MEKK	Aba Tibetan and Qiang autonomous prefecture	Markang county	—	—	2,571	102°14′3039″	31°53′3036″	2018.6.14

### Distribution and Resource Overview

The survey results showed that the *P. mira* in Sichuan was mainly distributed in Ganzi Tibetan Autonomous Prefecture, with the largest number in Derong County, Batang County, and Xiangcheng County, followed by Baiyu County, Yajiang County, Jiulong County, Muli County, Daocheng County, and Kangding City, and the least number in Yanyuan County and Markang City. The planting area of Sichuan Province is about 2,800 mu, and the annual yield of *P. mira* is about 40 tons. The *P. mira* in Derong County is mainly distributed in the Jinsha River and its tributaries in the Dingqu River basin, namely, Ancient Township, Guxue Township, Bari Township, Ciwu Township, Rilong Township, Baisong Township, Xulong Township, and Sizha Township. There are about 16,000 plants, among which there are about 500 mu of artificial cultivation. Trees of wild *P. mira* are older, more than 30 years old, and most of them reach 100 years old. In the past, there were many trees of *P. mira*, but some trees of wild *P. mira* have been cut down due to busy farming, unmanned management, land development, and other reasons. The study group found that reserves of wild *P. mira* were very large in Derong County, and this may be related to the fact that Derong County is the second-to-the-last county in China with access to highways, which has resulted in little attention from scientists, slow economic development, and relatively little deforestation of *P. mira*. The trees of *P. mira* in Batang County are both wild and planted. Trees of wild *P. mira* are mainly distributed in Zhongzheng Town, Changbo Township, and Zhubalong Township, while the generally distributed villages and towns are Diwu Township, Zhongxinrong Township, Yarigong Township, Xia Qiong Town, Suwalong Township, Lawa Township, Dangba Township, and Moduo Township. The villages and towns without *P. mira* distribution are Bomi Township, Bogoxi Township, Lieyi Township, Deda Township, Cuola Township, Chaluo Township, Songduo Township, and Jiaying Township. Along the G215 line along the Jinsha River, *P. mira* is rarely seen due to human activities, but they can be seen in Mangkang City, Changdu, Tibet. Batang County government accords great importance to *P. mira* planting and has planned to build a planting base in Zhongza town. The trees of *P. mira* in the township are all wild, concentrated along the road from township to Daocheng County, mainly in Qingde Township, Qingyi Township, Zhengdou Township, Shuiwa Township, Shagong Township, etc. The counties with general distribution of *P. mira* are Jiulong County, Baiyu County, Kangding City, Yajiang County, Daocheng County, and Muli County. There are many trees of *P. mira* in Char Village and the nearby semi-alpine mountains in Jiulong County, so Char Village is also known locally as “Tao hua cun”. The *P. mira* of Kangding city is mainly distributed in Pusharong Township and Jiju Township. The *P. mira* in Baiyu County is mainly wild, and it is distributed in 11–12 townships in Baiyu County, mainly including Magong Township, Zhangdu Township, Gaiyu Township, Shanyan Township, Shama Township, and beside the Jinsha River, especially near the Laser tunnel in Zhangdu Township, Dingqu River ditch, Rejia Township, and also kang Basi below until Jinsha River. There are about 4,000 plants of *P. mira* in Yajiang County, which have not been harvested and used in recent years. They are mainly distributed in villages and towns along the Yalong River, such as Murong Township, Xiala Township, Bajiao Lou Township, Hekou Town, Milong Township, Malangco Township, Egu Township, Bayirong Township, Poshihe Township, and Yanyihe Township. *P. mira* in Daocheng County is mainly distributed in Chitu Township, Shangrila Township, Dongyi District, and Mengzi Township, all of which are wild *P. mira*, without artificial planting or pest control. A large number of natural populations of *P. mira* were found in Muli County (Yang, 1989), but after investigation, it was found that the reserves had been greatly reduced, mainly because a large number of trees of *P. mira* were cut down and planted with other crops with more economic value, and it is also very likely that this investigation team has not yet entered the original forest for investigation. Our research group investigated Liziping Township, Boke Township, Qiaowa Township, Kerr Township, and other towns in Muli County, and there was no *P. mira* distribution; only three to five *P. mira* was sporadically distributed along National Highway 616, which only flowered but did not bear fruit. Beside the road, more crops or fruits with greater economic value are planted, such as *Prunus armeniaca* L., *Juglans regia* L., and *Prunus persica* (L.) Batsch. In the course of investigation, a villager surnamed Luo in Muli County reported that there were trees of *P. mira* in front and behind each house in seven villages in Kala Township of Muli County. The trees were of uneven age. Among them, there were about 110 households in Maolao Village, two to five trees per household, and a total of about 500 plants. There are sporadic *P. mira* growing in Lugu Lake Town, Yanyuan County, and the hanging fruit is very few and the fruit is small. There are few *P. mira* in Markang County, which are less distributed in the residential buildings along the banks of the river, and sporadically distributed in the nearby mountains. Information on resource distribution was also obtained during the interview. During the visit to the herbal medicine market in Yongquan Street, Xichang, Liangshan Yi Autonomous Prefecture, it was found that there was no kernel of *P. mira* for sale in the market, but according to merchants in the market, there was *P. mira* distribution in Yuexi County. However, due to the long distance of Yuexi County, the field investigation was not carried out. In addition, according to a staff member of the Health Bureau in Xinlong County, Ganzi Tibetan Autonomous Region, *P. mira* is distributed in Heping Township, Dagai Township, Dong’an Township, Sewei Township, Asequ Gou Township, Jialaxi Township, Pigao Township, Zituoxi Township, Rulong Town, Youlaxi Township, Mayue Township, and Luozuo Township in Xinlong County. It is also assumed that *P. mira* is distributed in Danba County, Mao County, Dege County, Jinchuan County, Wenchuan County, Xiaojin County, Luhuo County, Heishui County, and Li County. The grassland-dominated counties may have no *P. mira* distribution, including Shiqu County, Ganzi County, Seda County, Rangtang County, and Litang County. These data provided reference for the follow-up investigation on the distribution of resources of *P. mira*.

### Artificial Planting and Seedling Rising

At present, there are three *P. mira* seedling and planting bases. First, the planting base of Derong County in Sichuan Province is located in Rilong Village and Riyong Village of Ciwu Township, covering an area of about 100 mu. Second, the planting base of Batang County in Sichuan Province is in Wave Village, Zhongza Town, with an area of about 1700 mu and Renmian Village with an area of about 600 mu. If 55 plants are planted per mu, there are about 120,000 plants planted. Batang County and Derong County seedlings were mainly from Benzilan Town, Deqin County, Yunnan Province. Third, the *P. mira* seedlings in Benzilan Town, Deqin County, Yunnan Province are about 10,000 plants, with an average height of 1.5 m, with a nutrition bowl and growing well. The seeds of *P. mira* need to be soaked in clean water for 4–7 days and disinfected with potassium permanganate; farm manure, especially sheep dung, is added to the soil, and then two to three nuts are poured into the nutrition bowl, which is beneficial to absorb the water and nutrition of the lower soil, and the nutrition bowl is placed in the greenhouse to be watered accordingly to prevent livestock and insect pests. The seedlings of *P. mira* are sown in January every year, and the seedlings of 2 years are the best for transplanting. At present, the main problems in planting bases are due to topography and climate, serious shortage of water resources, and weak water conservancy facilities, which affect the normal growth of artificial *P. mira* forest.

### Economic Value and Medicinal Value

*P. mira* can be used as both medicine and food, but there is less in-depth development and utilization at present, as shown in Table 4. The pulp is mainly used to feed livestock (though a few farmers and herdsmen will eat it), the core is mainly sold to medicinal herbs, and the kernel can be used as medicine. During the visit, it was found that the villagers in Yajiang County would eat the dried kernel raw or fry it, and during hard times, they would pick *P. mira*, take out the kernel, mash it, and add it into butter tea (to remove the bitter taste) before eating it. In the past, the kernel of *P. mira* from Derong County, Batang County, and Xiangcheng County was mainly sold to herbal medicine merchants in Shangri-La city, and eventually went to the “*He hua chi* (Lotus Pond)” Chinese herbal medicine market in Chengdu. The price of peach cores was 7–14 yuan/kg, and the price difference of peach kernel was 30–60 yuan/kg. Because the *P. mira* has not been part of the “*Pharmacopoeia of the People*’*s Republic of China*”, it affects the scope of application and usage. At present, due to the reduction of the purchase volume, farmers and herdsmen mainly use the *P. mira* for feeding pigs, cattle, and other livestock, a small part for sale. Most farmers believe that the fruit of *P. mira* tastes sour, and they occasionally pick it to quench their thirst, but our research group tried to deep fry and stir fry the kernel of *P. mira* and found that it tasted good. After freezing the flesh with sugar, the flesh has an “original peach flavor”, a crisp taste, and a suitable sour and sweet taste, and is edible.

**TABLE 4:** Statistics of the use of *P. mira* in Ganzi Tibetan Autonomous Prefecture, Sichuan Province.

County	Part used	Usage	The yield of core (tons)	The unit price of core (RMB/kg)	The yield of kernel (tons)	The unit price of kernel (RMB/kg)	Peasant household income (RMB/year)
Derong County	Pulp, core	The pulp was fed to pigs and the core was sold	100–130	7–13	10	50–60	5,000–15,000
Batang County	Pulp, kernel	The pulp was fed and the kernel was used as medicine and sold	60	10–30	15	60	3,000–10,000
Xiangcheng County	Pulp	the pulp was fed to pigs and cows	—	14	7	30	150–500
Daocheng County	Core	The core was sold	4	14	1	45	—
Kangding County	Core	The core was sold	—	14	5	30	—
Yajiang County	Pulp	The pulp was fed	—	—	—	—	—
Baiyu County	Pulp	The pulp was fed to pigs	—	—	—	—	—

## Discussion

**FIGURE 3:**
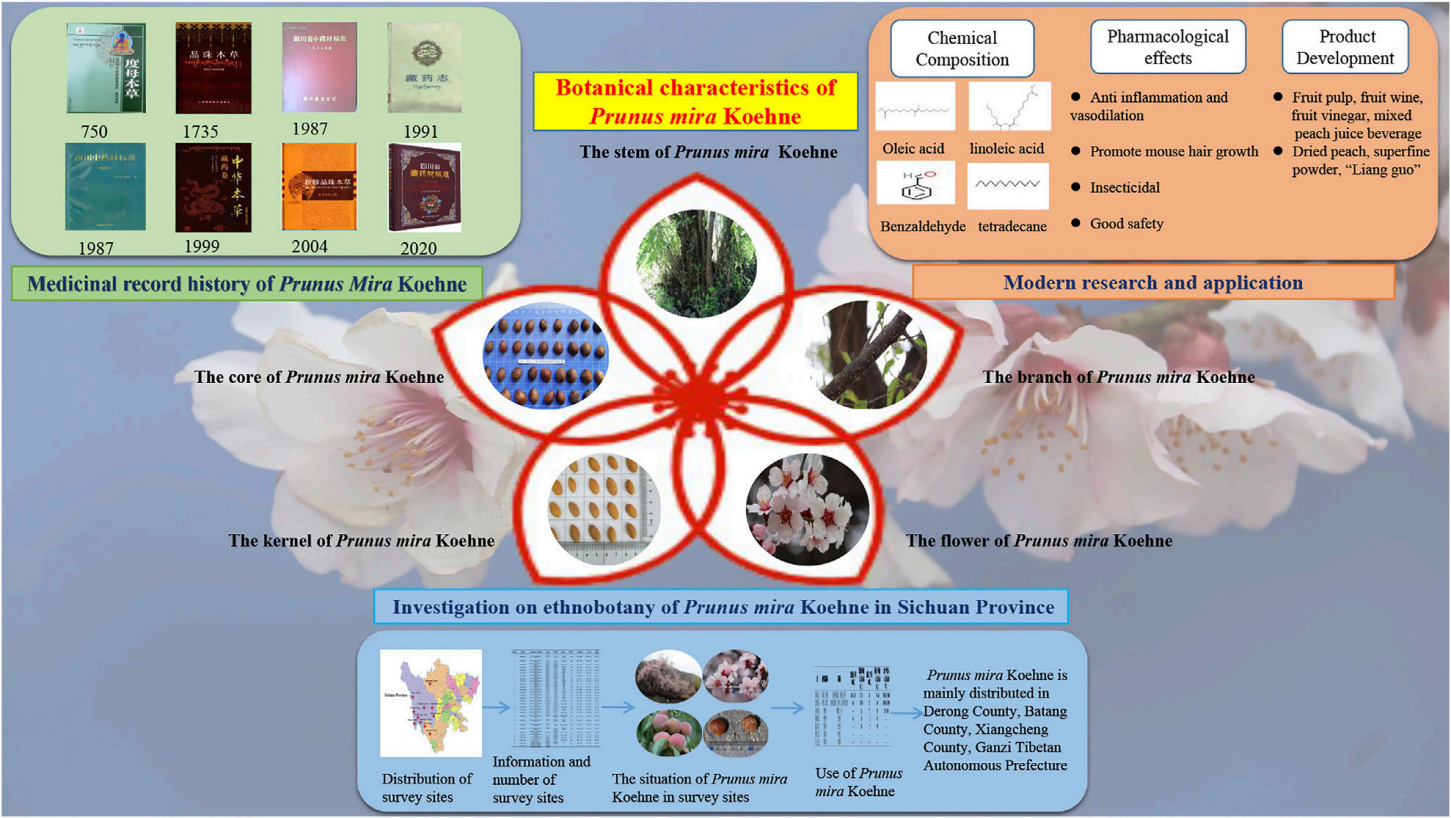
Investigation and evaluation of *P. mira*.

*P. mira* was first recorded in *Dumu Materia Medica*, marking its presence in history after 1,300 years. From its modern application, it has been proven to be medicinal and edible. Its pulp is treated to have edible value. The fruit of *P. mira* is low in sugar, and high in minerals and vitamin C (Zeng et al., 2009); thus, its market potential is huge and has been growing in the low-sugar preserved fruit (Cai et al., 1997a), juice (Cai et al., 2002), fruit wine (Zhong et al., 2012), and fruit vinegar (Kan et al., 2018) market. Pharmacological studies have found that it has anti-inflammatory effects and can expand blood vessels (Xu and Xu, 1997); it can also promote hair growth (Zhou et al., 2020). Researchers visited and investigated some districts and counties of Ganzi Tibetan Autonomous Prefecture, Liangshan Yi Autonomous Prefecture, and Aba Tibetan and Qiang Autonomous Prefecture in Sichuan. The aim is to understand the resource distribution, growth environment, and application value of *P. mira*. It is found that due to social changes, man-made logging, farmland reclamation, and other factors, the main distribution and quantity have greatly changed compared with the recorded data. At present, *P. mira* is mainly distributed in Ganzi Tibetan Autonomous Prefecture, and the largest number of *P. mira* is found in Derong County, Batang County, and Xiangcheng County. As shown in Figure 3, the local county government has gradually begun to pay attention to the utilization of resources and industrial development of *P. mira*, and established the planting base of *P. mira*. However, county-level funding is low and it may be difficult to support the investment. We can consider introducing social capital to adopt the form of equity for investment and income distribution. In addition, other features of *P. mira* are also worthy of attention. Its longevity and cold resistance characteristics can bring great genetic value, and it plays an important role and value in maintaining the genetic diversity of peaches and cultivating long-lived and cold-resistant peach varieties (Zhang et al., 2013).

In Sichuan Province, the kernels of *P. mira* were used as medicine of semen persicae in the market. Because *P. mira* is not included in the *Pharmacopoeia of the People’s Republic of China*, its application scope and usage are affected. Semen persicae in Chinese Pharmacopoeia come from *Prunus persica* (L.) Batsch and *Prunus davidiana* (Carrière) Franch (Pharmacopoeia, 2015). It is distributed in Hebei, Yunnan, Gansu, Sichuan, and other provinces (Yang et al., 2018). At present, HPLC was used to compare the fingerprints of the kernel of *P. mira* and other semen persicae, and it was found that their chemical constituents were generally similar. It provides some basis for the mixed use of the kernel of *P. mira* and other semen persicae in the market (Du et al., 2019). The similarities and differences of the two herbs should be evaluated by quality control indicators (Li et al., 2019), safety evaluation, and system effectiveness evaluation. If *P. mira* can be used as one of the *Pharmacopoeia of the People’s Republic of China* semen persicae source, it can greatly promote the development of *P. mira* harvest, sales, and downstream industries.

Natural ecology is the basis of local culture, which restricts the material conditions of daily life. If the environment and its cultural ecology are well handled, and the continuous accumulation of intangible capital and a virtuous cycle are ensured, the popularization and sustainable development of local knowledge depend on the development and interaction of academic achievements (Wang, 2020). From the perspective of China’s consumption trend development, tourism consumption accounts for an increasing proportion of people’s income. The way people travel from single to diversified development, rural tourism, agricultural tourism, and folk tourism were becoming more and more popular among tourists (Liu, 2007). Because the flower of *P. mira* has a certain ornamental value, local government could develop tourism. The local government can use the “combination of medicine and tourism” as the development idea. At the same time, it can use the help of the development plan of the “11th Five-Year Plan” and “Tourism Planning” (Province, 2017) of Sichuan Province to vigorously develop the tourism industry. Nyingchi in Tibet has had high-quality ecological resources linked by the flowers of *P. mira* as a link since ancient times, and the flowers of *P. mira* tourism and cultural festival that has gradually arisen attracts a large number of tourists every year (Luo and Wang, 2019). Nyingchi in Tibet makes full use of the superior resources of the flower of *P. mira* to develop tourism and improve economy. High-quality “ecological” resources are the biggest advantage of Tibetan ecotourism, and ecotourism is the cornerstone of sustainable tourism (Guo, 1997).

Quality control and value chains should be ensured for such an important herb. There is a need to popularize this plant by government, national, internal, social, and media agencies to increase people’s awareness of *P. mira* and protect its identity and quality. Data regarding many aspects of this plant such as mechanisms of action and pharmacological effects of specific active components are still limited. Additional clinical studies should be conducted in the future. The scientific community should also develop a more active role in developing its research, conservation, and cultivation strategies.
